# Nathan Shoemaker

**Published:** 1868-07

**Authors:** 


Died, in Philadelphia, on the 11th of June, Nathan Shoemaker, M. D., aged 80 years. Dr. S. had for some years retired from the active duties of the profession, but at one time he enjoyed an extensive practice, and was universally respected for his skill and high moral character.

